# ATP/P2X7 receptor signaling as a potential anti-inflammatory target of natural polyphenols

**DOI:** 10.1371/journal.pone.0204229

**Published:** 2018-09-24

**Authors:** Erika Nuka, Kohta Ohnishi, Junji Terao, Yoshichika Kawai

**Affiliations:** 1 Department of Food Science, Graduate School of Biomedical Sciences, Tokushima University, Tokushima, Japan; 2 Faculty of Nursing and Rehabilitation, Konan Women's University, Hyogo, Japan; Karolinska Institutet, SWEDEN

## Abstract

Innate immune cells, such as macrophages, respond to pathogen-associated molecular patterns, such as a lipopolysaccharide (LPS), to secrete various inflammatory mediators. Recent studies have suggested that damage-associated molecular patterns (DAMPs), released extracellularly from damaged or immune cells, also play a role in the activation of inflammatory responses. In this study, to prevent excess inflammation, we focused on DAMPs-mediated signaling that promotes LPS-stimulated inflammatory responses, especially adenosine 5’-triphosphate (ATP)-triggered signaling through the ionotropic purinergic receptor 7 (P2X7R), as a potential new anti-inflammatory target of natural polyphenols. We focused on the phenomenon that ATP accelerates the production of inflammatory mediators, such as nitric oxide, in LPS-stimulated J774.1 mouse macrophages. Using an siRNA-mediated knockdown and specific antagonist, it was found that the ATP-induced enhanced inflammatory responses were mediated through P2X7R. We then screened 42 polyphenols for inhibiting the ATP/P2X7R-induced calcium influx, and found that several polyphenols exhibited significant inhibitory effects. Especially, a flavonoid baicalein significantly inhibited ATP-induced inflammation, including interleukin-1β secretion, through inhibition of the ATP/P2X7R signaling. These findings suggest that ATP/P2X7R signaling plays an important role in excess inflammatory responses and could be a potential anti-inflammatory target of natural polyphenolic compounds.

## Introduction

Inflammation is an essential immune response that enables survival during infection or injury and maintains tissue homeostasis. Under noxious or abnormal conditions, several inflammatory mediators, including cytokines, chemokines, reactive gases, and eicosanoids, produced by immune cells attract other leukocytes to the injured and/or infected tissues to exclude foreign substances through phagocytosis, killing effects, and cell death induction [1–3]. Therefore, inflammation is important for host defense or tissue remodeling. However, abnormal inflammation with over or persistent expression of mediators could lead to the initiation and progression of various diseases, such as type 2 diabetes [4], cardiovascular diseases [5–7], neurodegenerative diseases [8,9], and cancers [10]. Inflammation is triggered not only by pathogen-associated molecular pattern molecules (PAMPs) derived from microorganisms, such as a lipopolysaccharide (LPS), but also by damage-associated molecular patterns (DAMPs), either actively or passively released from damaged or immune cells, such as uric acid crystals, nucleotides, and the chromatin-associated protein HMGB1. Recent studies have suggested that DAMPs could be implicated in excessive inflammation which plays a key role in the pathogenesis of several diseases, and therefore targeting DAMPs or their receptors might be beneficial [11,12]. In addition, Yang *et al*. reported the importance of therapeutic approaches to selectively target DAMPs-mediated inflammation while preserving physiological protective immune responses, such as PAMPs-elicited inflammatory responses [13].

Adenosine-5’-triphosphate (ATP) is present at high levels (5–8 mM) in the cytoplasm for cellular homeostasis [14,15], and is released through multiple pathways, including the membrane degradation of necrotic cells, connexin hemichannels from immune cells, pannexin hemichannels from apoptotic cells [16], lysosomal exocytosis from dying cells depending on autophagy [17], and vesicular exocytosis from epithelial cells and osteoblasts and nerves [14]. It is suggested that released extracellular ATP has important and diverse effects on many biological processes including inflammasome formation, promoting cytokine release, pain, defective phagocytic clearance, smooth muscle contraction, and neurotransmission [18–20]. These effects are mediated by plasma membrane purinergic receptors comprised of ATP-gated ionotropic P2Xs and guanine nucleotide-binding protein-coupled P2Ys. On the other hand, many researchers have pointed out that extracellular ATP, released from injured or damaged cells under abnormal conditions, causes undesirable responses to the surrounding cells. For example, the ATP levels of bronchoalveolar lavage fluid are increased by smoking, and the extracellular ATP contributes to the pathogenesis of chronic obstructive pulmonary disease [21], lung fibrosis [22], and allergic asthma [23]. Studies using knockout or antagonists of the ionotropic purinergic receptor 7 (P2X7R), preferentially expressed in immune cells, suggested that P2X7R activation by extracellular ATP is linked to colitis [24], systemic lupus erythematosus and rheumatoid arthritis [25], coronary artery diseases [26], short survival during infection [27], and the progression of brain tumors [28]. Recent findings also suggest that P2X7R-mediated ATP signaling may represent a potential therapeutic target to improve health and that natural products or synthetic compounds inhibiting P2X7R could be useful in clinical trials [29,30].

Many reports have suggested that natural polyphenols, present in vegetables or fruits, have potential health-beneficial effects including anti-inflammatory effects through a variety of biological activities, such as antioxidant and radical scavenging activities, regulation of intracellular signaling cascades or transcriptional networks, and improvements of the endothelial structure or function [31,32]. It has been reported that natural polyphenols down-regulate PAMPs-induced inflammatory mediators, such as LPS-induced pro-inflammatory cytokines, both *in vitro* and *in vivo* [32,33]. Toll-like receptor 4 (TLR4) and other pathogens recognition receptors-induced mitogen-activated protein kinases (MAPKs) and nuclear factor κB (NF-κB) pathways were shown to be important targets for polyphenols, such as quercetin [34], epigallocatechin gallate (EGCG) [35], luteolin [36], apigenin [37], and others [38,39]. However, it still remains unknown whether DAMPs-mediated excess inflammatory responses could be the target of natural compounds including polyphenols.

In this study, to develop a new strategy for the prevention of inflammatory diseases, we investigated the molecular actions of extracellular ATP on the LPS-induced macrophage inflammation and carried out screening of natural polyphenols for the inhibition of ATP signaling. We propose that P2X7R-mediated ATP signaling could be a potential anti-inflammatory target of natural polyphenols.

## Materials and methods

### Reagents

Dulbecco's modified eagle medium (DMEM, 4.5 g/l glucose, liquid), penicillin-streptomycin mixed solution (Stabilized), ATP disodium salt hydrate from yeast, adenosine-5'-diphosphate (ADP) sodium salt from a bacterial source, adenosine-5'-monophosphate (AMP) sodium salt from yeast, apyrase from potato, protease inhibitor cocktail, phosphatase inhibitor cocktail, bovine serum albumin (BSA, F-V), and 3-(4,5-dimethyl-2-thiazolyl)-2,5-diphenyltetrazolium bromide (MTT) were obtained from Nacalai Tesque, Inc. (Kyoto, Japan). Fetal bovine serum (FBS) and LPS from *Escherichia coli* O127:B8 were obtained from the Sigma-Aldrich (St. Louis, USA). DMEM without phenol red (4.5 g/l glucose, liquid), sulfanilamide, *N*-(1-naphthyl)ethylenediamine dihydrochloride, sodium nitrite, and rabbit IgG were obtained from Wako Pure Chemical Industries (Osaka, Japan). Rabbit polyclonal antibody to inducible nitric oxide synthase (iNOS) (sc-650) and mouse monoclonal antibody to β-actin (sc-47778) were obtained from Santa Cruz Biotechnology, Inc. (Texas, USA). Rabbit polyclonal antibody to P2X7R was obtained from GeneTex, Inc. (LA, USA). Rabbit monoclonal antibodies to phospho-signal transducer and activator of transcription (STAT) 1 (Tyr701) (58D6), phospho-interferon regulatory factor (IRF)-3 (Ser396) (4D4G), phospho-p38 MAPK (Thr180/Tyr182) (D3F9) XP, phospho-p44/42 MAPK (Erk1/2) (Thr202/Tyr204) (D13.14.4E) XP, rabbit polyclonal antibody to phospho-stress-activated protein kinases (SAPKs)/c-jun N-terminal kinases (JNKs) (Thr183/Tyr185), and mouse monoclonal antibody to inhibitor κB (IκB)-α were obtained from Cell Signaling Technology, Inc. (MA, USA). Rabbit polyclonal antibody to mouse interferon (IFN)-β for neutralization was obtained from Pestka Biomedical Laboratories, Inc. (NJ, USA). BAPTA-AM was obtained from Invitrogen (CA, USA). Fluo 4-AM was obtained from Dojindo Laboratories (Kumamoto, Japan). 4-(*N*,*N*-Dipropylsulfamoyl) benzoic acid (probenecid) was obtained from Tokyo Chemical Industry Co., Ltd. (Tokyo, Japan). Pluronic F-127 was obtained from Life Technologies Japan, Ltd. (Tokyo, Japan). All other chemicals were commercially available analytical grade reagents. TAK-242 was obtained from Merck.

### Cell culture

Murine macrophage-like J774.1 cells were obtained from American Tissue Culture Collection, and cultured in DMEM, which contains glucose (4.5 mg/ml) and L-glutamine (584 μg/ml), supplemented with 10% heat-inactivated FBS, penicillin (100 units/ml), and streptomycin (100 μg/ml) at 37°C under 5% CO_2_.

### Transfection of siRNA

Stealth RNAi siRNA against P2X7R (P2rx7 MSS276199) and a negative control (low GC) were obtained from Invitrogen (CA, USA). The transfection of siRNA was performed using the Neon Transfection System (Life Technologies Japan, Ltd., Tokyo, Japan). J774.1 cells (2 × 10^6^ cells) were resuspended in 100 μl resuspension buffer R containing 50 nM siRNA against P2X7R or control siRNA and transfected in 100 μl Neon tip using two pulses (1720 V input pulse voltage, 10 ms input pulse width). The transfected cells were seeded into 24-well plates (5 ×10^5^ cells/well) and cultured in antibiotic-free DMEM for 24–36 h, then used for each experiment.

### Measurement of nitric oxide production

Nitric oxide (NO) production was evaluated by measuring the metabolite nitrite (NO_2_^-^) using the Griess assay. J774.1 cells were seeded into 48-well plates and cultured overnight. After washing with phosphate-buffered saline (PBS), the medium was replaced with phenol red-free DMEM containing LPS (1 μg/ml) and adenosine analogs (1 mM). After incubation for 24 h, each cultured medium (100 μl) was mixed with 100 μl of Griess reagent [1% (w/v) sulfanilamide in 5% (w/w) phosphoric acid and 0.1% (w/v) *N*-(1-naphthyl)ethylenediamine dihydrochloride in water]. The optical density was then measured at 550 nm. The nitrite levels were calculated using a standard curve derived from authentic sodium nitrite.

### Western blotting

J774.1 cells were seeded into 24-well plates and cultured overnight. After treatment with each agent for the indicated times, the cells were washed with PBS and lysed with a radio-immunoprecipitation assay buffer [50 mM Tris-HCl (pH 7.4), 150 mM NaCl, 0.25% deoxycholic acid, 1% NP-40, 0.1% sodium dodecyl sulfate (SDS), 1 mM ethylenediaminetetraacetic acid] supplemented with both protease and phosphatase inhibitor cocktails. The protein concentrations were determined by a Protein Assay Bicinchoninate Kit (Nacalai Tesque, Kyoto, Japan). The protein samples were denatured by heating at 95°C for 5 min in the presence of SDS (2%) and 2-mercaptoethanol (5%), then separated using 10% SDS-polyacrylamide gels followed by a semi-dry blotting onto a polyvinylidene difluoride membrane (Hybond P, GE Healthcare). After blocking with EzBlock Chemi (Atto Corp., Tokyo, Japan) in Tris-buffered saline containing 0.05% Tween 20 (TTBS) at room temperature for 30 min, the membrane was washed with TTBS, then incubated overnight with a primary antibody (1:1000) in TTBS containing 5% BSA at 4°C. After washing, the membrane was incubated with horseradish peroxidase-conjugated secondary antibody (1:2000) in TTBS containing 5% skim milk at room temperature for 1 h. After washing, chemiluminescent detection was performed using Chemi-Lumi One L or Chemi-Lumi One Super (Nacalai Tesque, Kyoto, Japan) and visualized by Ez-Capture MG (Atto Corp., Tokyo, Japan).

### Real-time reverse transcription-polymerase chain reaction

The total RNA was extracted from the cells using Sepasol-RNA I Super G (Nacalai Tesque, Kyoto, Japan). cDNA was synthesized using ReverTra Ace qPCR RT Master Mix with gDNA Remover (Code No. FSQ-301, Toyobo Co., Ltd., Osaka, Japan). PCR amplification was performed with THUNDERBIRD SYBR qPCR Mix (Code No. QPS-201, Toyobo Co., Ltd., Osaka, Japan) using the LightCycler Nano System (Roche Diagnostics K.K., Tokyo, Japan). The relative expression of each mRNA was calculated according to the 2^-*ΔΔ*Ct^ method and normalized to the glyceraldehyde-3-phosphate dehydrogenase (GAPDH) expression. The primer sequences are listed in the S1 Table.

### Measurement of calcium influx

Cells were seeded into 48-well plates and cultured overnight. After washing with PBS, the medium was replaced with phenol red-free DMEM containing Fluo 4-AM (5 μg/ml), Pluronic F-127 (0.02%), and probenecid (1.25 mM). After incubation for 1 h, the medium was replaced with phenol red-free DMEM containing each polyphenol (50 μM) and probenecid (1.25 mM). After incubation for 10 min, an equal volume of phenol red-free DMEM containing ATP (4 mM) and the corresponding polyphenol (50 μM) was added to each well. After incubation for 10 min, the cell plates were scanned using Typhoon FLA 9500 at the following settings: 473 nm blue LD laser, BPB1 emission filter, 500 V photomultiplier, 100-μm pixel size. The fluorescence intensity of each well was measured using Image Quant TL ver. 8.1.

### Cytotoxicity assay

Cells were seeded into 48-well plates and cultured overnight. After each stimulation, the medium was replaced with DMEM containing MTT (0.2 mg/ml) and incubated for 30 min. After removing the medium, the formazan was dissolved in dimethyl sulfoxide (200 μl/well) and the optical density was measured at 550 nm. The relative cell viability was calculated using the following formula: relative cell viability (%) = optical density of experimental groups / optical density of the control group × 100.

### ELISA for murine interleukin-1β

The amount of interleukin-1β (IL-1β) was determined according to the recommended protocol for the ELISA Development Kit for murine IL-1β (Peprotech, USA). Colorimetric detection was performed using the TMB Substrate Reagent Set (BD Biosciences, USA). The reaction was terminated by adding 2N sulfuric acid and the optical density was measured at 450 nm.

### Statistics

The data represent means ± S.D. of triplicate determinations. All the statistical analyses were performed by Tukey-Kramer method. The asterisks indicate statistical significance (*P* < 0.05).

## Results

### ATP accelerates NO production in LPS-stimulated J774.1 mouse macrophages

**Fig 1:**
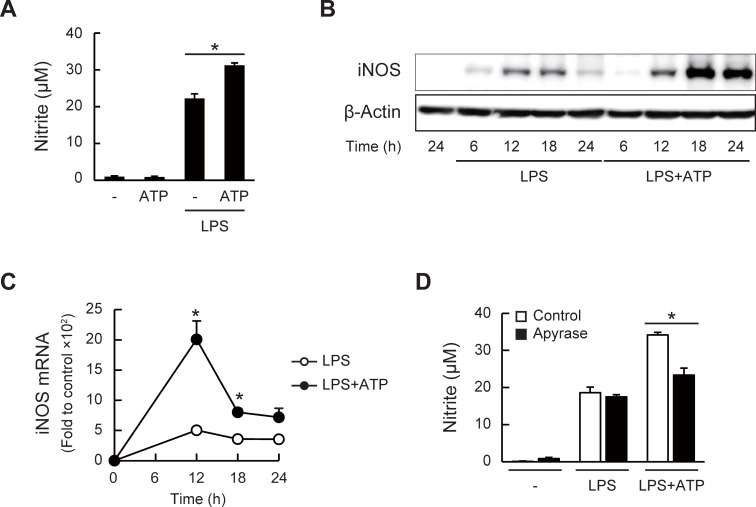
ATP accelerates LPS-induced NO production and iNOS expression. (A) NO production in J774.1 cells was evaluated by measuring the metabolite nitrite (NO_2_^-^) in culture supernatants using the Griess assay. The cells were treated with LPS (1 μg/ml) in the presence or absence of ATP (1 mM) for 24 h (*P < 0.05). (B) iNOS protein expression in LPS/ATP-treated cells was analyzed by western blotting. β-Actin was detected as the internal control. (C) Relative expression of iNOS mRNA measured by quantitative RT-PCR. GAPDH was evaluated as the normalization control (*P < 0.05 vs LPS-treated group). (D) Effect of apyrase (0.5 units/ml) on LPS/ATP-stimulated NO production (24 h after stimulation, *P < 0.05). (A, C, and D) Data represent means ± S.D. of triplicate determinations.

To examine the effects of ATP on the macrophage inflammation, J774.1 cells were treated with LPS in the presence or absence of ATP for 12–36 h. We observed that the LPS-induced NO production was enhanced after 24 h in the presence of ATP, although NO production was not induced by ATP alone (Fig 1A and Figure A in S1 Fig). The cell viability was not affected by the ATP (Figure B in S1 Fig). Both the protein and mRNA expressions of iNOS, a key enzyme generating NO, were also up-regulated by the addition of ATP (Fig 1B and 1C), but not by ATP alone (Figure C in S1 Fig). Given that the extracellular ATP is rapidly degraded by ectonucleotidases, such as CD39 [40,41], we investigated whether the NO production could certainly be enhanced by ATP, or the degradation products. As shown in Fig 1D, the enhanced NO production by ATP was abolished by the treatment with apyrase, an enzyme that hydrolyzes ATP into AMP. We also confirmed that ADP and AMP did not enhance the LPS-induced NO production (Figure D in S1 Fig).

### ATP facilitates IFN-β expression to enhance the NO production

**Fig 2:**
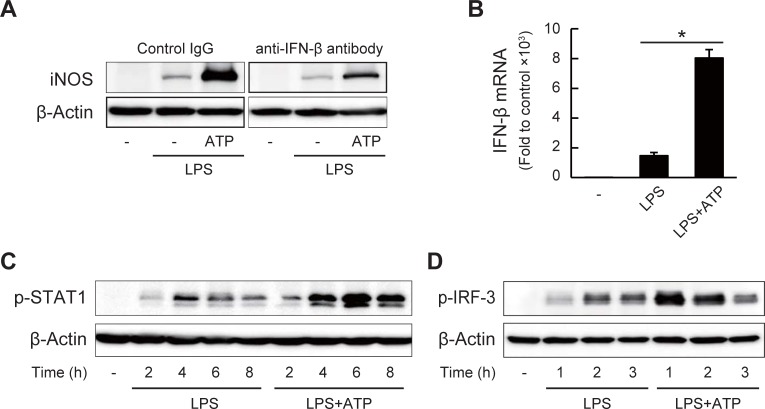
ATP facilitates LPS-induced IFN-β expression leading to NO production. (A) Effects of neutralizing antibody to IFN-β on iNOS protein expression in J774.1 cells treated with LPS (1 μg/ml) and ATP (1 mM) for 12 h. Lanes were noncontiguous but from same blot membrane at same time. (B) Relative expression of IFN-β mRNA in LPS/ATP-treated cells (3 h) measured by quantitative RT-PCR. Data represent means ± S.D. of triplicate determinations (*P < 0.05). (C) Phosphorylation of STAT1 in LPS/ATP-treated cells after 2–8 h. (D) Phosphorylation of IRF-3 in LPS/ATP-treated cells after 1–3 h.

To investigate the molecular mechanisms of the ATP-enhanced NO production, we analyzed the upstream signaling pathway regulating the iNOS transcription. As it is reported that secreted interferons are necessary for full expression of the iNOS mRNA [42,43], we evaluated the involvement of IFN-β, expressed strongly in J774.1 cells, in the LPS/ATP-induced expression of iNOS. As shown in Fig 2A, the addition of anti-IFN-β neutralizing antibody almost completely suppressed the LPS-induced iNOS protein expression in both presence and absence of ATP, suggesting the involvement of autocrine/paracrine signaling of IFN-β in LPS-induced iNOS expression. Indeed, consistent with the induction of iNOS, ATP enhanced the LPS-induced IFN-β mRNA expression and phosphorylation of STAT1, downstream of the IFN-β signaling (Figs 2B and 2C). In addition, the phosphorylation of IRF-3, the essential transcription factor of IFN-β [44], was also up-regulated by the addition of ATP (Fig 2D).

### P2X7R activation up-regulates IFN-β expression and NO production

**Fig 3:**
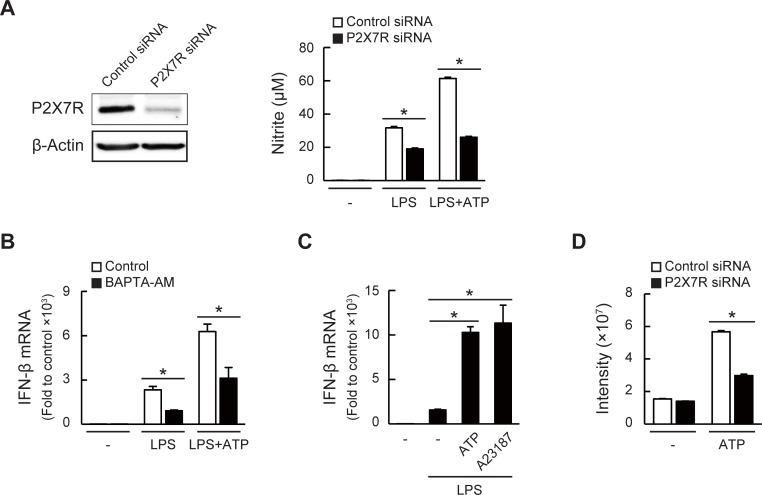
P2X7R activation and following Ca^2+^ influx up-regulate IFN-β expression and NO production. (A) Left, P2X7R protein expression was analyzed 24 h after transfection of control or P2X7R-specific siRNA. Right, NO production in transfected cells was evaluated using Griess assay after stimulation with LPS (1 μg/ml) in the presence or absence of ATP (1 mM). (B) Effects of BAPTA-AM (20 μM) pre-treatment (1 h) on LPS/ATP-induced IFN-β mRNA expression (3 h after stimulation). (C) Effects of A23187 (10 μM) on LPS/ATP-induced IFN-β mRNA expression (3 h after stimulation). (D) Ca^2+^ influx in control or P2X7R knockdown cells was measured using Fluo 4 fluorescence at 10 min after ATP (2 mM) stimulation. All the data represent means ± S.D. of triplicate determinations (*P < 0.05).

It has been reported that P2X7R, expressed on most immune cells, such as macrophages, is activated through millimolar concentrations of extracellular ATP (0.1–4 mM) and participates in inflammation and pain mechanisms [30,45–48]. Therefore, we next examined the involvement of P2X7R in the enhancement of the LPS-induced inflammation by ATP. As shown in Fig 3A, LPS-induced NO production, especially in the presence of ATP, was significantly reduced in the P2X7R knockdown cells, showing the effects of ATP through P2X7R activation. Since the activated P2X7R has been shown to facilitate the influx of extracellular cations, including calcium ions (Ca^2+^) [48,49], we next investigated the roles of the Ca^2+^ influx in the LPS/ATP-induced IFN-β expression. As shown in Fig 3B, BAPTA-AM, an intracellular Ca^2+^ chelator, significantly inhibited the LPS/ATP-induced IFN-β expression. In addition, A23187, a Ca^2+^ ionophore, also reproduced the enhancing effect of ATP on the IFN-β expression (Fig 3C). We confirmed that the Ca^2+^ influx, monitored using Fluo 4-AM, was induced by the treatment of ATP for 10 min and was abrogated in the P2X7R knockdown cells (Fig 3D). These results strongly suggest that the ATP/P2X7R-mediated Ca^2+^ influx contributes to the LPS/ATP-induced IFN-β expression.

### P2X7R-mediated ATP signaling affects TLR4 signaling to enhance IFN-β expression and NO production

**Fig 4:**
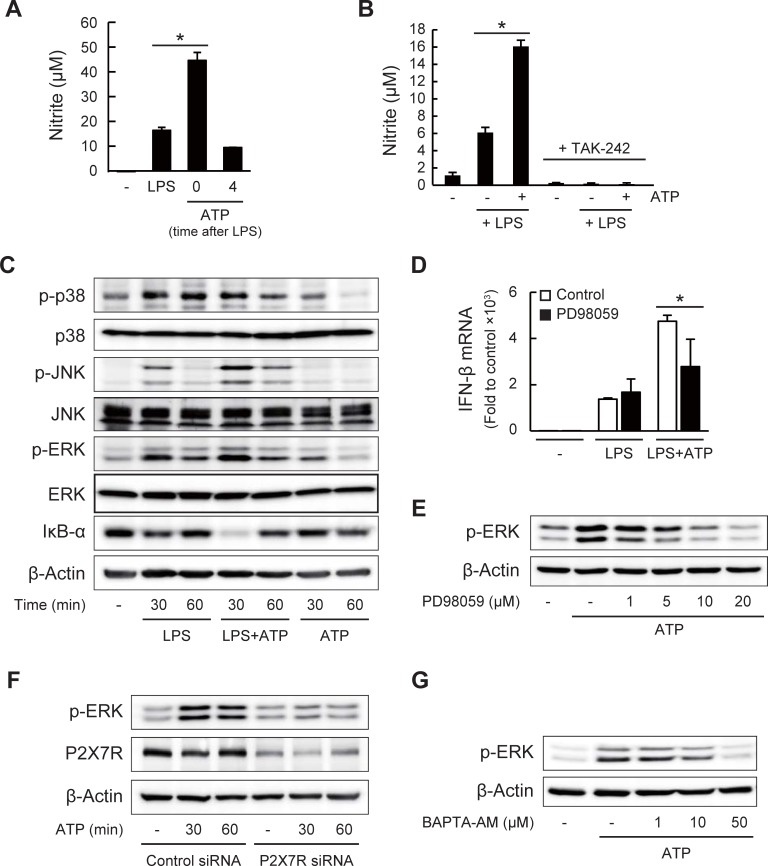
P2X7R-mediated ATP signaling affects LPS/TLR4 signaling. (A) Effects of co- and post-treatment with ATP on LPS-induced NO production in J774.1 cells. ATP (1 mM) was added at 0 or 4 h after LPS (1 μg/ml) treatment. NO production was evaluated by Griess assay 24 h after LPS treatment (*P < 0.05). (B) Effects of a TLR4 inhibitor TAK-242 (5 μM) on LPS/ATP-induced NO production in J774.1 cells (*P < 0.05). (C) Phosphorylation of p38, JNK, and ERK, as well as the total proteins, and IκB-α degradation in LPS/ATP-treated cells were analyzed by western blotting. (D) Effects of PD98059 (20 μM) pre-treatment (1 h) on LPS/ATP-induced IFN-β mRNA expression 3 h after stimulation (*P < 0.05). (E) Effects of PD98059 (pre-treatment for 1 h) on ATP-induced ERK phosphorylation (1 h after ATP stimulation). (F) Effects of P2X7R knockdown on ATP-induced ERK phosphorylation. Cells transfected with control or P2X7R-specific siRNA were stimulated with ATP (1 mM). (G) Effects of BAPTA-AM (pre-treatment for 30 min) on ATP-induced ERK phosphorylation. (A, B and D) Data represent means ± S.D. of triplicate determinations.

Next, to understand the actions of ATP on enhanced inflammation, we examined the timing of the ATP stimulation for enhancing LPS-induced NO production. As shown in Fig 4A, we found that the LPS-induced NO production was enhanced only when cells were co-treated with LPS and ATP, but hardly affected when ATP was added a few hours after the LPS stimulation. This result suggested that the P2X7R-mediated ATP signaling stimulated upstream of the LPS-induced TLR4 signaling. As shown in Fig 4B, ATP-enhanced NO production in LPS-treated cells was not observed in the presence of a specific TLR4 inhibitor TAK-242, showing the effects of ATP on the TLR4 signaling. We then investigated whether ATP could activate the LPS/TLR4 signaling pathways, such as NF-κB and MAPKs pathways. As shown in Fig 4C, the LPS-induced degradation of IκB-α and phosphorylation of ERK and JNK at 30 min were up-regulated by ATP. Phosphorylation of p38 was not affected by ATP. The phosphorylation of ERK was also induced by ATP alone. It was reported that the activation of these pathways results in the activation of the downstream transcription factors, such as NF-κB and activator protein-1, to induce the IFN-β and iNOS mRNA expression [42,43,50]. In addition, it has been reported that the P2X7R activation induces ERK phosphorylation via Ca^2+^-dependent or -independent activation of the upstream kinases [51–53]. We then investigated the involvement of the ERK phosphorylation on the effects of ATP. As shown in Fig 4D, PD98059, an inhibitor of the MEK1/2-ERK pathway, attenuated the enhancing effects of ATP on the IFN-β expression. It was also confirmed that the ATP-induced ERK phosphorylation was indeed inhibited in the presence of PD98059 (Fig 4E). Furthermore, the ATP-induced ERK phosphorylation was reduced in the P2X7R knockdown cells and also inhibited by BAPTA-AM (Fig 4F and 4G). These results suggest that ATP sensing by P2X7R and the following Ca^2+^ influx and ERK phosphorylation may be potential pathways for enhancing the LPS-induced IFN-β expression, leading to the increased iNOS expression and NO production.

### Inhibitory effects of polyphenols on ATP/P2X7R signaling

**Fig 5:**
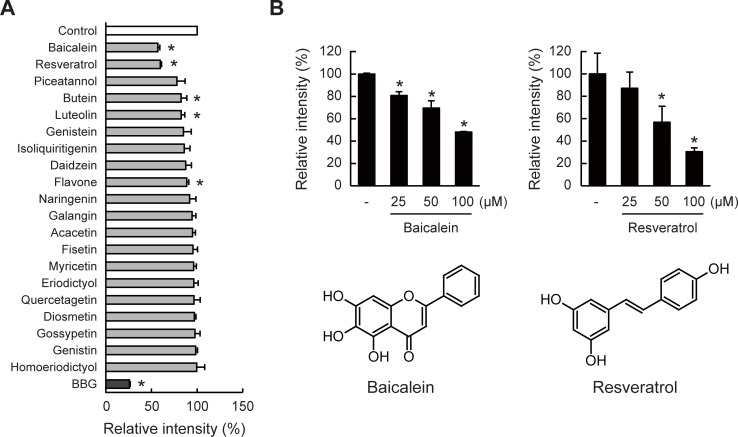
Inhibitory effects of polyphenols on ATP/P2X7R-mediated Ca^2+^ influx. (A) J774.1 cells were pre-treated with each polyphenol (50 μM) or BBG (2 μM) for 10 min followed by ATP stimulation (2 mM) for 10 min. Relative Fluo 4 fluorescence intensities (%) to control (stimulated with ATP alone) were calculated. Data for polyphenols that exhibited inhibitory effects (below 100%) are shown in the graph. (B) Dose-dependent inhibition of ATP-induced Ca^2+^ influx by baicalein (left) and resveratrol (right). Chemical structures of baicalein and resveratrol are also illustrated. All the data represent means ± S.D. of triplicate determinations (*P < 0.05 vs control).

We next investigated the inhibitory effects of natural polyphenols on the ATP/P2X7R signaling by measuring the Ca^2+^ influx as an indicator of the P2X7R activation. Among the 42 polyphenols (listed in S2 Table), we found that, as well as brilliant blue G (BBG), a P2X7R antagonist [54], baicalein and resveratrol exhibited the most significant inhibitory effects on the ATP-induced Ca^2+^ influx (Fig 5A). It was also confirmed that these two polyphenols inhibited the P2X7R activation in dose-dependent manners (Fig 5B). On the other hand, inhibitory effects of these polyphenols on the ATP-induced ERK phosphorylation could not be observed (Figure A in S2 Fig). This may be due to the fact that these polyphenols induced ERK phosphorylation regardless of the presence or absence of ATP. These results suggest that specific polyphenols could target the ATP/P2X7R signaling, probably by antagonizing ATP and suppressing the Ca^2+^ influx.

### Anti-inflammatory effects of baicalein through interfering ATP/P2X7R signaling

**Fig 6:**
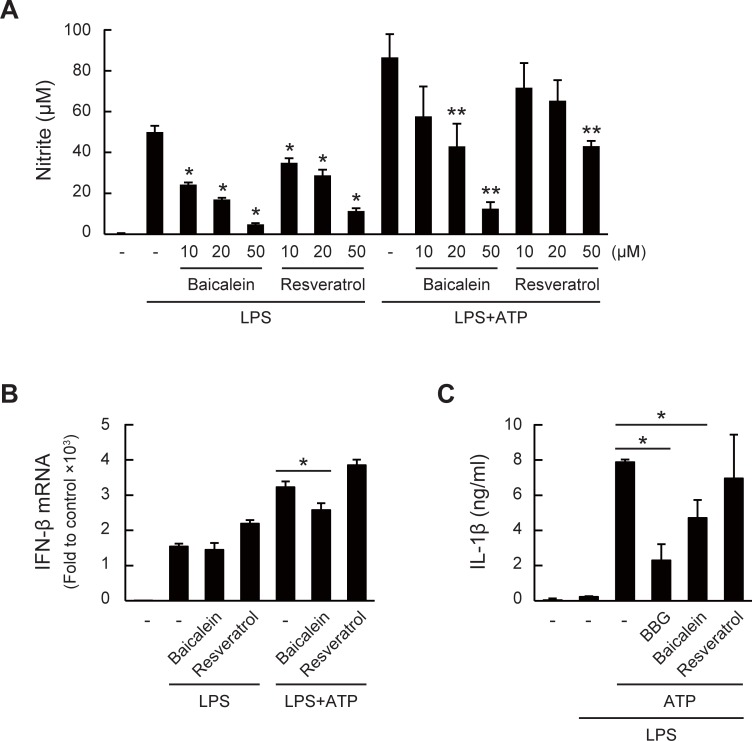
Anti-inflammatory effects of baicalein through interfering ATP/P2X7R signaling. (A) Dose-dependent effects of baicalein and resveratrol on LPS/ATP-induced NO production. Cells were pre-treated with each polyphenol for 1 h followed by LPS/ATP stimulation for 24 h (*P < 0.05 vs. LPS group and **P < 0.05 vs. LPS + ATP group). (B) Effects of baicalein and resveratrol (10 μM pre-treatment for 1 h) on LPS/ATP-induced IFN-β mRNA expression 3 h after stimulation (*P < 0.05). (C) Effects of baicalein and resveratrol on ATP-triggered IL-1β secretion in LPS-stimulated J774.1 cells. Cells were treated with LPS (1 μg/ml) for 4 h followed by the addition of ATP (5 mM). Each polyphenol (50 μM) or BBG (10 μM) was added 30 min before the ATP treatment (*P < 0.05). All the data represent means ± S.D. of triplicate determinations.

The results shown above indicated that ATP facilitates inflammatory responses through P2X7R, and that several polyphenols, such as baicalein and resveratrol, inhibit ATP/P2X7R signaling. We next investigated whether these polyphenols could indeed attenuate inflammation through inhibiting ATP/P2X7R signaling. Pre-treatment of baicalein or resveratrol inhibited the LPS/ATP-induced NO production in dose-dependent manners (Fig 6A). We confirmed that these polyphenols did not affect the cell viability under this experimental condition (Figure B in S2 Fig). As reported in many papers, polyphenols could inhibit the LPS/TLR4 signaling [32,38]. Therefore, the inhibitory effects of these polyphenols on NO production were conceivably due to the inhibition of not only the ATP/P2X7R signaling but also the LPS/TLR4 signaling. Thus, to investigate whether the inhibition of the ATP/P2X7R signaling by these polyphenols definitely confers the anti-inflammatory effects, we examined the effects of baicalein and resveratrol on the IFN-β expression, more upstream than the NO production. As shown in Fig 6B, baicalein inhibited the ATP-enhanced IFN-β expression at a lower concentration that did not affect the expression induced by LPS alone, whereas resveratrol rather induced IFN-β expression under this condition.

To further propose that the polyphenols could attenuate the ATP/P2X7R signaling, we then examined the inhibitory effects of baicalein and resveratrol on the secretion of IL-1β, an LPS-induced pro-inflammatory cytokine, the production of which is tightly regulated and specifically triggered by ATP as secondary stimuli [55]. As shown in Fig 6C, IL-1β was hardly detectable in cultured supernatants of the cells treated with the vehicle and LPS alone. On the other hand, the secretion of IL-1β was significantly induced upon ATP treatment 4 h after the LPS treatment. The secretion was significantly inhibited in the presence of BBG, showing that ATP sensing by P2X7R specifically contributes to IL-1β secretion. Under this experimental condition, the IL-1β secretion was significantly inhibited by pre-treatment with baicalein for 10 min before the ATP stimulation. On the other hand, similar to the results on IFN-β expression, resveratrol did not inhibit the IL-1β secretion. These results demonstrated that baicalein could be a potential candidate as a natural inhibitor of the ATP/P2X7R signaling.

## Discussion

Inflammation is an important defense response in noxious conditions, such as infection, whereas abnormal inflammatory responses are shown to cause various diseases. LPS-induced inflammation is a well-studied model of infection and diseases, therefore, the molecular signaling through the TLR4 receptor could be a target for therapy. Recent studies have shown that released endogenous molecules from damaged or immune cells, called DAMPs, are also implicated in the activation of inflammatory responses. In this study, we investigated the molecular actions of extracellular ATP, considered as one of the prototypical DAMP molecules [56,57], on the LPS-induced macrophage inflammation. Based on the molecular actions of ATP, to develop a new strategy for the prevention of inflammatory diseases, we evaluated the inhibitory effects of natural polyphenols on ATP signaling. We first observed that NO production and iNOS expression in the LPS-stimulated J774.1 macrophages were enhanced by ATP (Fig 1A–1C). It has been reported that the extracellular ATP is rapidly degraded to ADP and AMP, whereas the LPS-induced NO production was not enhanced by these degradation products (Figure D in S1 Fig). Although we did not examine the actual enzyme activity of ectonucleotidases in J774.1 cells, we scarcely detected the degradation products of ATP by a liquid chromatography-tandem mass spectrometry in culture supernatants of J774.1 treated with 1 mM ATP, strongly suggesting that the effects of ATP in this study reflect the action of ATP itself. NO has been recognized not only as a vasodilator and a neurotransmitter, but also as anti-microbial, anti-tumor, tissue-damaging, and immune-modulating products in the immune system. NO plays a dual role during inflammation by exerting protective and toxic effects in parallel, and this dual effect has been explained as being dependent on the NO concentration [58–60]. Although we did not investigate the phenotypic consequences of the up-regulated NO production, we speculated that LPS/ATP-enhanced NO production might lead to disorders and diseases associated with excess inflammation.

We then investigated the activation of the transcription factors and signal transduction pathways involved in the LPS/ATP-induced iNOS expression. We observed that the LPS-induced IFN-β expression and the downstream STAT1 phosphorylation were up-regulated by ATP. STAT1 has been reported to be an essential transcription factor for the iNOS expression [43]. In addition, we found that ATP facilitated the LPS-induced TLR4 signaling, such as the NF-κB and MAPK pathways, and the phosphorylation of IRF-3, the transcription factor for IFN-β. TLR4 signaling is generally separated into the myeloid differentiation primary response gene 88 (MyD88)-dependent pathway for expression of the pro-inflammatory cytokines and the TIR domain-containing adaptor protein-inducing IFN-β (TRIF)-dependent pathway for expression of the Type I IFNs. It has been suggested that NF-κB and MAPK could be activated through both pathways and that IRF-3 could be through the TRIF-dependent pathway [61,62]. Our results indicated that ATP accelerated both pathways, leading to the activation of the iNOS transcription factors. We also found that the mRNA expression of other LPS-induced inflammatory mediators, such as tumor necrosis factor-α (TNF-α), cyclooxygenase-2 (COX-2), IL-6, IL-10, and IL-1β, were also affected in the presence of ATP (S3 Fig). These results suggested that ATP accelerated upstream pathways in the LPS/TLR4 signaling resulting in perturbation of the downstream mRNA expression of iNOS and other inflammatory mediators.

We focused on the involvement of P2X7R regarding the effects of ATP because P2X7R is highly expressed in immune cells and responds to higher ATP concentrations. Using the siRNA-induced knockdown and antagonist of P2X7R, we demonstrated that P2X7R is indeed involved in the ATP-enhanced inflammatory responses. Our results that the LPS-induced NO production without ATP addition was also reduced in the P2X7R knockdown cells (Fig 3A) suggest the activation of the P2X7R signaling during stimulation with only LPS. Although similar observations that ATP or the P2X7R agonist enhanced LPS-induced inflammatory responses including IFN-β expression have been reported [63,64], our results clearly revealed the molecular actions of ATP through P2X7R, leading to enhanced NO production. For example, we demonstrated that ATP stimulated the Ca^2+^ influx and activated intracellular signaling, such as ERK phosphorylation, which subsequently enhanced the expression of the LPS-induced IFN-β expression and NO production. In addition, the LPS/ATP-enhanced IFN-β expression was reduced by BAPTA-AM and PD98059 (Figs 3 and 4), suggesting that the intracellular Ca^2+^ and the activation of MEK/ERK are both important for the LPS/ATP-induced inflammatory signaling. It has been reported that ATP binding to the extracellular loop of P2X7R results in not only channel opening but also structural and/or functional changes of the intracellular proteins or membrane lipids, such as calmodulin, heat shock proteins, cytoskeletal proteins, and phosphatidylinositol 4,5-phosphate, all of which bind to the C-terminal of P2X7R [65–67]. Therefore, these observations suggest that both the Ca^2+^-dependent and -independent signals of P2X7R are potential pathways in the ATP-induced inflammatory regulation.

Many studies, including the pre-clinical studies, have suggested that P2X7R could be therapeutically important targets because of the involvement in inflammatory diseases and pain. Although various negative or positive modulators of the P2X7R activation including natural products, synthetic molecules, and monoclonal antibodies, have been demonstrated, there is no approved medicine in clinical use [30,68–70]. In addition, little is known about the beneficial effects of dietary compounds targeting P2X7R [29]. Therefore, to prevent ATP/P2X7R-mediated abnormal inflammation, we explored the inhibitory effects of polyphenols, the largest group of phytochemicals, on the ATP/P2X7R signaling. We then found that a flavonoid baicalein (5,6,7-trihydroxyflavone) and a stilbenoid resveratrol significantly inhibited the ATP/P2X7R-mediated Ca^2+^ influx (Fig 5). We further investigated the effects of baicalein and resveratrol on the LPS/ATP-induced inflammation, and found that, as expected, these polyphenols indeed inhibited the LPS/ATP-induced NO production (Fig 6A). However, at least under this experimental condition, we could not distinguish whether these polyphenols modulated the LPS/TLR4 signaling or ATP/P2X7R signaling because many reports have demonstrated the inhibitory effects of polyphenols on the TLR4 signaling. For instance, resveratrol inhibits the kinase activity of TANK binding kinase 1 associating with TRIF, which is the TLR4 downstream component, resulting in decreasing the NF-κB and IRF-3 activation [71,72]. Another study also reported that quercetin inhibits the Src- and Syk-mediated PI3K phosphorylation and subsequent TLR4/MyD88/PI3K complex formation, resulting in the reduced activation of downstream signaling [73]. Therefore, to elucidate that the inhibition of the ATP/P2X7R signaling by baicalein or resveratrol definitely contributes to the anti-inflammatory or therapeutic effects, we evaluated the effects of these polyphenols on IL-1β secretion. In culture experiments, IL-1β is neither matured nor secreted by the treatment with only LPS. It is generally recognized that ATP addition after a few hours of LPS stimulation triggered inflammasome activation leading to the formation and secretion of the matured IL-1β [18,55]. We then examined the effects of two polyphenols on the ATP-triggered IL-1β secretion and found that the IL-1β secretion was significantly inhibited by baicalein, but unfortunately, not by resveratrol (Fig 6C). Although we did not investigate the activation of inflammasome in this study, the result strongly suggested that baicalein reduced the maturation and secretion of IL-1β through inhibition of the ATP/P2X7R signaling. Effects of antagonizing ATP/P2X7R signaling by baicalein or other natural products on inflammatory responses *in vivo* have not yet been determined within this study. Utilization of P2X7R-deficient mice in the near future will clarify the involvements of ATP in inflammatory responses and the possible interactions between natural polyphenols and P2X7R *in vivo*.

Baicalein and its glucuronide (baicalin), the major components of *Scutellaria baicalensis* Georgi, a popular herb used in traditional Chinese herbal medicine, are shown to prevent cancer, inflammation, cardiovascular diseases, and hypertension [74]. He *et al*. reported that baicalein inhibited TLR4 expression, NF-κB, and MAPK signal pathways and subsequently reduced the levels of pro-inflammatory cytokines [75]. Our current results also proposed a new health-beneficial activity of baicalein through inhibition of ATP/P2X7R signaling. Although the inhibitory effect of baicalin was not examined in this study, we previously reported that the glucuronides of flavonoids could be deconjugated into the aglycones by macrophages, resulting in increased anti-inflammatory activity as compared to the parent glucuronides [76]. Therefore, interaction between macrophages and baicalin could result in the inhibitory effect on the ATP/P2X7R signaling, similar to the action of the aglycone baicalein. On the other hand, in spite of the inhibitory effect on ATP-induced Ca^2+^ influx, resveratrol did not inhibit ATP-enhanced IFN-β expression and IL-1β secretion (Fig 6B and 6C). Resveratrol rather induced LPS/ATP-induced IFN-β expression. Although we predicted that both baicalein and resveratrol could also inhibit the ATP/P2X7R-mediated ERK phosphorylation, as well as Ca^2+^ influx, these polyphenols, especially resveratrol, conversely enhanced the ATP-induced ERK phosphorylation (Figure A in S2 Fig). Similarly, several papers also demonstrated the ERK activation by polyphenols [77–80]. Within this study, it is still unclear how tight the link between Ca^2+^ influx and ERK phosphorylation is in the ATP signaling. Resveratrol, found in grape products such as red wine, has been shown to improve cardiovascular performance and extend the lifespans of non-mammals and mice [81]. However, we could not conclude that, at least from our observations, resveratrol is a potent inhibitor of ATP/P2X7R signaling.

Overall, our results indicated the potential preventive effects of baicalein on exacerbating the ATP/P2X7R signaling, not only LPS signaling, under noxious or abnormal conditions. We demonstrated the molecular actions of the extracellular ATP through P2X7R on the LPS signaling, resulting in excess inflammation, and also proposed a new strategy for the prevention of excess inflammation by inhibiting the ATP/P2X7R signaling using natural polyphenols, such as baicalein. As well as ATP/P2X7R, involvements of other pairs of DAMPs/receptors such as advanced glycation end-product/RAGEs and β-amyloid, in the inflammatory responses have been suggested [82–83]. Therefore, our conclusion is that the ATP/P2X7R signaling and other DAMPs signaling could be a new potential target of drugs and natural compounds including polyphenols for the prevention of disorders and diseases associated with excess inflammation.

## Supporting information

S1 Fig Effects of ATP on NO production and cell viability. (A) NO production in J774.1 cells was evaluated using the Griess assay. Cells were treated with LPS (1 μg/ml) in the presence or absence of ATP (1 mM) for the indicated time periods (*P < 0.05 vs LPS-treated group). (B) Cell viability (%) was analyzed by cytotoxicity assay after LPS/ATP stimulation at the indicated time points. (C) Protein and mRNA expression of iNOS in LPS/ATP-stimulated J774.1 cells. Cells were treated with LPS (1 μg/ml) in the presence or absence of ATP (1 mM) or ATP alone for the indicated time periods. iNOS protein expression (β-actin as a loading control) was evaluated by immunoblotting. iNOS mRNA expression was determined by RT-qPCR (GAPDH as endogenous control, duplicate determinations). (D) Effects of ATP, ADP, and AMP on the LPS-induced NO production (*P < 0.05). All the data represent means ± S.D. of triplicate determinations.
(PDF)

S2 Fig Effects of baicalein and resveratrol on ATP-induced ERK phosphorylation and cell viability. (A) Dose-dependent effects of baicalein and resveratrol on ATP-induced ERK phosphorylation in J774.1 cells. Cells were pre-treated with each polyphenol for 1 h followed by ATP (1 mM) stimulation for 1 h. (B) Cell viability (%) was analyzed by cytotoxicity assay after treatment with each polyphenol for 24 h. Data represent means ± S.D. of triplicate determinations.
(PDF)

S3 Fig Effects of ATP on the expression of LPS-induced inflammatory mediators. Relative mRNA expression of TNF-α (A), COX-2 (B), IL-6 (C), IL-10 (D), and IL-1β (E) in J774.1 cells were measured by quantitative RT-PCR after LPS/ATP treatment. All the data represent means ± S.D. of triplicate determinations (*P < 0.05, A, vs LPS + ATP-treated group; B-E, vs LPS-treated group).
(PDF)

S1 Table Primer sequences used for quantitative RT-PCR. (PDF)

S2 Table Chemical structures and names of selected polyphenols used in this study. (PDF)
