# Supplementary figures and images for: ATP/P2X7 receptor signaling as a potential anti-inflammatory target of natural polyphenols
Source: PLoS One. 2018 Sep 24;13(9):e0204229. doi: 10.1371/journal.pone.0204229 (PMC6152980; doi:10.1371/journal.pone.0204229)

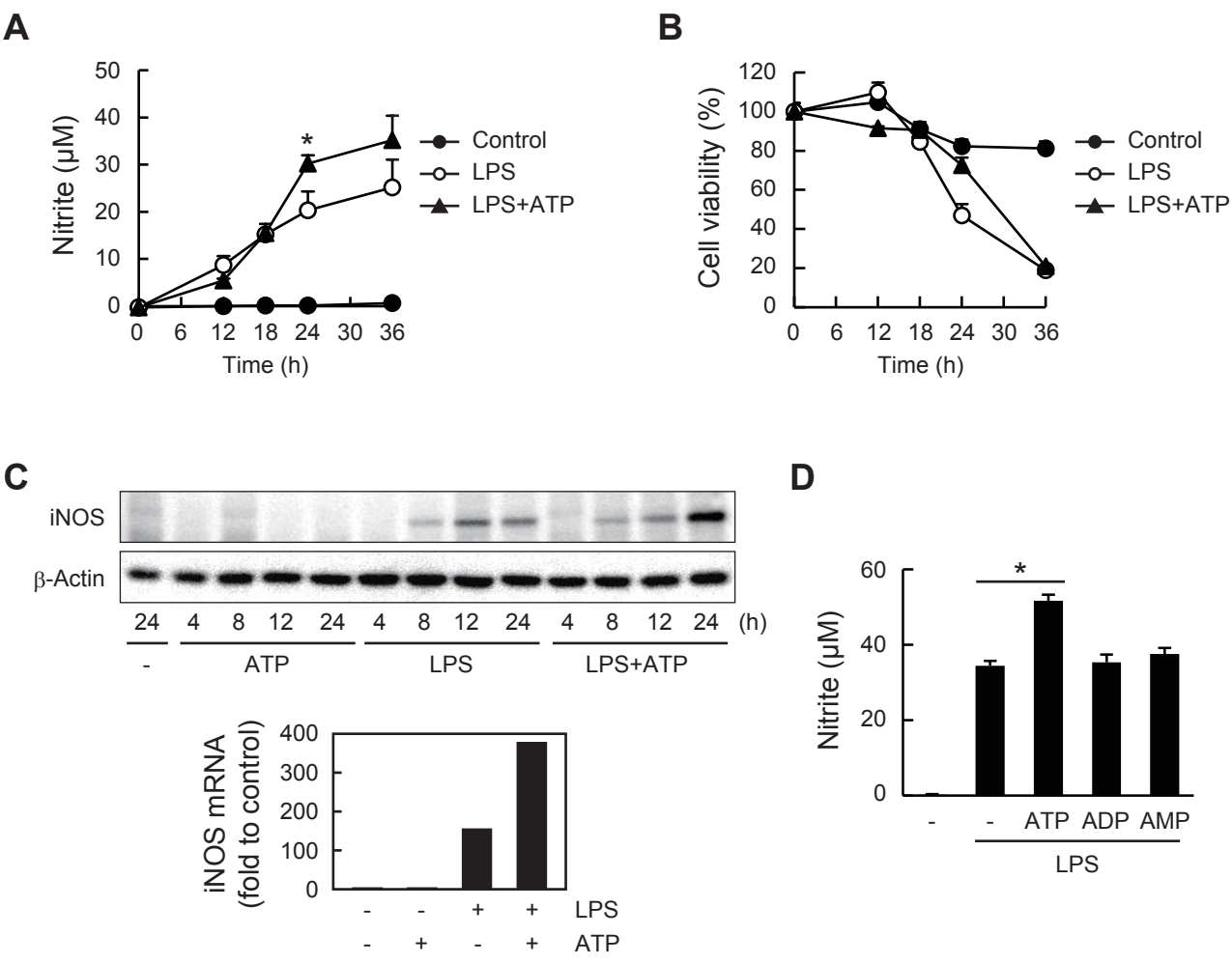

**S1 fig.**

Supplement: S1 Fig — (A) NO production in J774.1 cells was evaluated using the Griess assay. Cells were treated with LPS (1 μg/ml) in the presence or absence of ATP (1 mM) for the indicated time periods (*P < 0.05 vs LPS-treated group). (B) Cell viability (%) was analyzed by cytotoxicity assay after LPS/ATP stimulation at the indicated time points. (C) Protein and mRNA expression of iNOS in LPS/ATP-stimulated J774.1 cells. Cells were treated with LPS (1 μg/ml) in the presence or absence of ATP (1 mM) or ATP alone for the indicated time periods. iNOS protein expression (β-actin as a loading control) was evaluated by immunoblotting. iNOS mRNA expression was determined by RT-qPCR (GAPDH as endogenous control, duplicate determinations). (D) Effects of ATP, ADP, and AMP on the LPS-induced NO production (*P < 0.05). All the data represent means ± S.D. of triplicate determinations. (PDF) [file pone.0204229.s001.pdf]

**A**

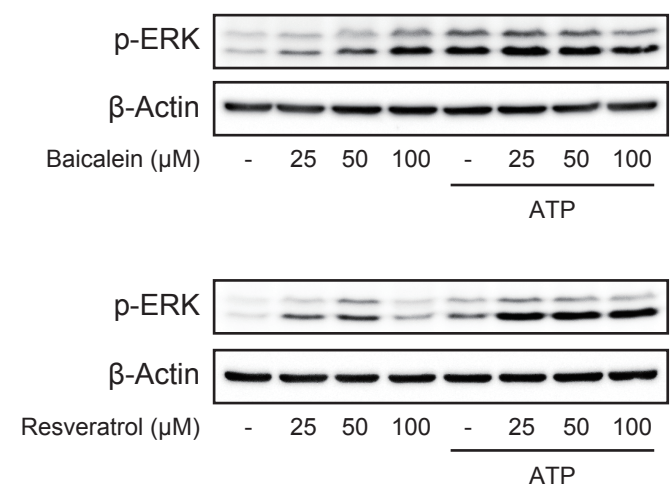

**B**

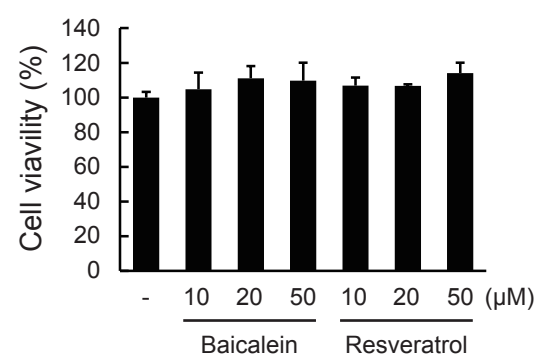

**S2 fig.**

Supplement: S2 Fig — (A) Dose-dependent effects of baicalein and resveratrol on ATP-induced ERK phosphorylation in J774.1 cells. Cells were pre-treated with each polyphenol for 1 h followed by ATP (1 mM) stimulation for 1 h. (B) Cell viability (%) was analyzed by cytotoxicity assay after treatment with each polyphenol for 24 h. Data represent means ± S.D. of triplicate determinations. (PDF) [file pone.0204229.s002.pdf]

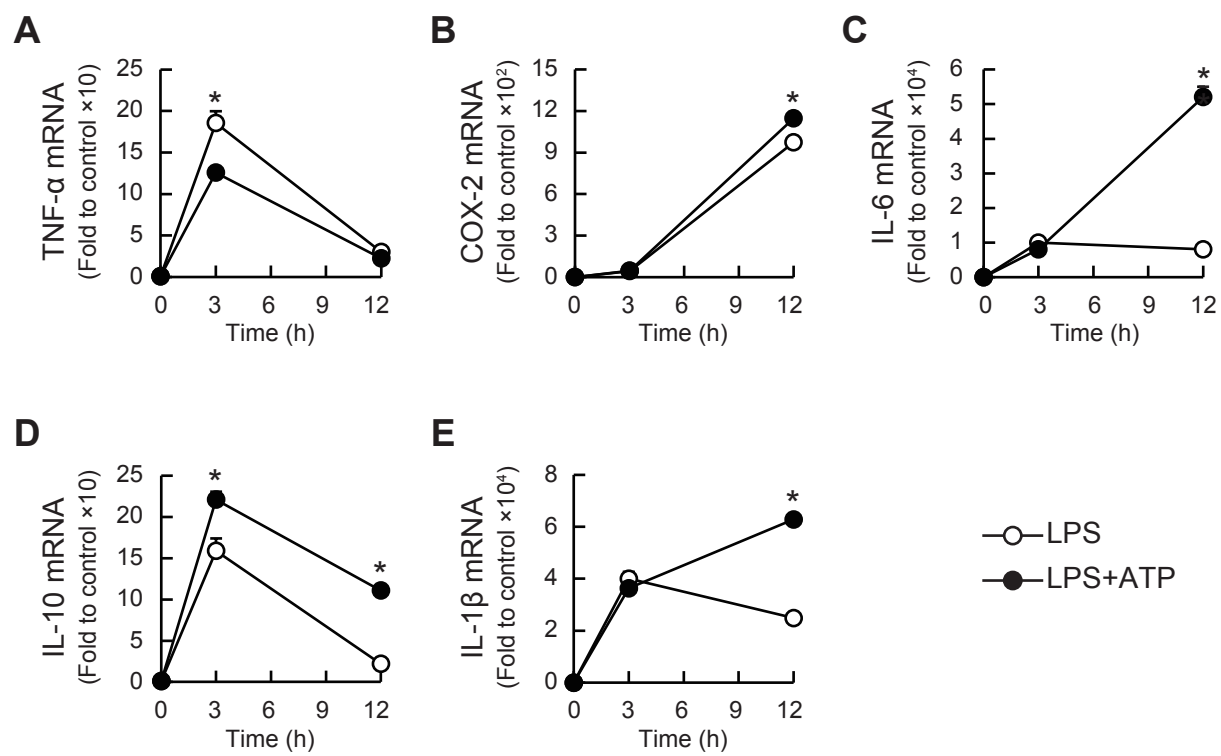

**S3 fig.**

Supplement: S3 Fig — Relative mRNA expression of TNF-α (A), COX-2 (B), IL-6 (C), IL-10 (D), and IL-1β (E) in J774.1 cells were measured by quantitative RT-PCR after LPS/ATP treatment. All the data represent means ± S.D. of triplicate determinations (*P < 0.05, A, vs LPS + ATP-treated group; B-E, vs LPS-treated group). (PDF) [file pone.0204229.s003.pdf]
