# Supplementary material for: ATP/P2X7 receptor signaling as a potential anti-inflammatory target of natural polyphenols
Source: PLoS One. 2018 Sep 24;13(9):e0204229. doi: 10.1371/journal.pone.0204229 (PMC6152980; doi:10.1371/journal.pone.0204229)
Supplement: S1 Table — (PDF) [file pone.0204229.s004.pdf]

S1 Table. Primer sequences used for quantitative RT-PCR

Gene	Primer	Sequence (5' to 3')
GAPDH	forward	TCAAGCTCATTTCCTGGTAT
	reverse	GTCCAGGGTTTCTTACTCCT
iNOS	forward	CAGCTGGGCTGTACAAACCTT
	reverse	CATTGGAAGTGAAGCGTTTCG
IFN-β	forward	AGCTCCAAGAAAGGACGAACAT
	reverse	GCCCTGTAGGTGAGGTTGATCT
TNF-α	forward	GTGGAACTGGCAGAAGAGGC
	reverse	AGACAGAAGAGCGTGGTGGC
COX-2	forward	TTGGGGAGACCATGGTAGAG
	reverse	GCTCGGCTTCCAGTATTGAG
IL-6	forward	ATCCAGTTGCCTTCTTGGGACTGA
	reverse	TAAGCCTCCGACTTGTGAAGTGGT
IL-10	forward	AGTTCAGAGCTCCTAAGAGAGTTGTGA
	reverse	CCTCTGAGCTGCTGCAGGAA
IL-1β	forward	AAATACCTGTGGCCTTGGGC
	reverse	CTTGGGATCCACACTCTCCAG
