# Supplementary material for: ATP/P2X7 receptor signaling as a potential anti-inflammatory target of natural polyphenols
Source: PLoS One. 2018 Sep 24;13(9):e0204229. doi: 10.1371/journal.pone.0204229 (PMC6152980; doi:10.1371/journal.pone.0204229)
Supplement: S2 Table — (PDF) [file pone.0204229.s005.pdf]

S2 Table. Chemical structures and names of selected polyphenols used in this study

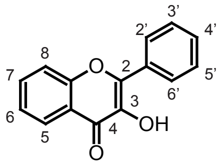

Flavonol

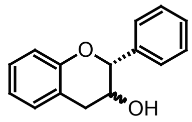

Flavanol

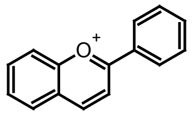

Anthocyanin

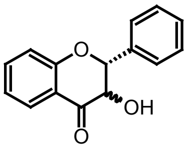

Flavanonol

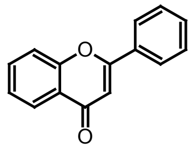

Flavone

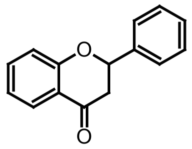

Flavanone

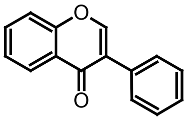

Isoflavone

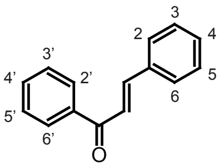

Chalcone

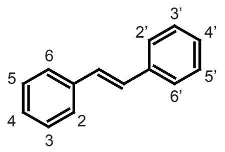

Stilbene

Class	Family	Name	Synonyms
Flavonoids	Flavonols	Isorhamnetin	3,4',5,7-Tetrahydroxy-3'-methoxyflavone
		Galangin	3,5,7-Trihydroxyflavone
		Gossypetin	3,3',4',5,7,8-Hexahydroxyflavone
		Tamarixetin	3,3',5,7-Tetrahydroxy-4'-methoxyflavone
		Myricetin	3,3',4',5,5',7-Hexahydroxyflavone
		Kaempferol	3,4',5,7-Tetrahydroxyflavone
		Quercetin	3,3',4',5,7-Pentahydroxyflavone
		Quercetagetin	3,3',4',5,6,7-Hexahydroxyflavone
		Rhamnetin	3,3',4',5-Tetrahydroxy-7-methoxyflavone
		Flavonol	3-Hydroxyflavone
		Morin	2',3,4',5,7-Pentahydroxyflavone
		Datiscetin	2',3,5,7-Tetrahydroxyflavone
		Fisetin	3,3',4',7-Tetrahydroxyflavone
	Flavanols	EC	3,3',4',5,7-Pentahydroxyflavane; (-)-Epicatechin
		ECg	(-)-Epicatechin 3-O-gallate
		EGC	(-)-Epigallocatechin
		EGCg	(-)-Epigallocatechin 3-O-gallate
	Anthocyanins	Cyanidin	3,3',4',5,7-Pentahydroxyflavylium
		Cyanidin 3-O-glucoside	Kuromanin
	Flavanonol	Taxifolin	3,3',4',5,7-Pentahydroxyflavanone
	Flavones	Flavone	2-Phenylchromone
		Baicalein	5,6,7-Trihydroxyflavone
		Diosmetin	3',5,7-Trihydroxy-4'-methoxyflavone
		Tricetin	3',4',5,5',7-Pentahydroxyflavone
		Apigenin	4',5,7-Trihydroxyflavone
		Acacetin	5,7-Dihydroxy-4'-methoxyflavone
		Chrysin	5,7-Dihydroxyflavone
		Luteolin	3',4',5,7-Tetrahydroxyflavone
	Flavanones	Eriodictyol	3',4',5,7-Tetrahydroxyflavanone
		Hesperetin	3',5,7-Trihydroxy-4'-methoxyflavanone
		Naringenin	4',5,7-Trihydroxyflavanone
		Homoeriodictyol	4',5,7-Trihydroxy-3'-methoxyflavanone
	Isoflavones	Genistein	4',5,7-Trihydroxyisoflavone
		Daidzein	4',7-Dihydroxyisoflavone
		Genistin	4',5,7-Trihydroxyisoflavone 7-O-glucoside
		Daidzin	4',7-Dihydroxyisoflavone 7-O-glucoside
	Chalcones	Butein	2',3,4,4'-Tetrahydroxychalcone
		Homobutein	2',4,4'-Trihydroxy-3-methoxychalcone
		Isoliquiritigenin	2',4,4'-Trihydroxychalcone
Stilbenes	Stilbenes	Resveratrol	3,4',5-Trihydroxystilbene
		Piceid	Resveratrol 3-O-glucoside
		Piceatannol	3,3',4,5'-Tetrahydroxystilbene
